# Predicting Immediate Marginal Milk Responses and Evaluating the Economics of Two-Variable Input Tactical Feeding Decisions in Grazing Dairy Cows

**DOI:** 10.3390/ani11071920

**Published:** 2021-06-28

**Authors:** Joanna W. Heard, Murray C. Hannah, Christie K. M. Ho, William J. Wales

**Affiliations:** 1 Department of Jobs, Precincts and Regions, Hamilton, VIC 3300, Australia; 2 Department of Jobs, Precincts and Regions, Ellinbank, VIC 3820, Australia; murray.hannah@agriculture.vic.gov.au (M.C.H.); bill.wales@agriculture.vic.gov.au (W.J.W.); 3 Department of Jobs, Precincts and Regions, Bundoora, VIC 3083, Australia; christie.ho@agriculture.vic.gov.au; 4 Centre for Agricultural Innovation, Faculty of Veterinary and Agricultural Sciences, School of Agriculture and Food, The University of Melbourne, Parkville, VIC 3010, Australia

**Keywords:** profitable, supplementary feeding, pasture, concentrates, forages

## Abstract

**Simple Summary:**

In Australia, feeding grazing dairy cows concentrate and forage supplements is common. Dairy farmers face the challenge of profitably feeding their cows in situations where there is significant variation in feed costs and milk price. We used the results of grazing experiments to develop equations that predict the yield of milk fat and milk protein when different combinations of concentrates and pasture + forage are fed to grazing lactating dairy cows. We applied economic principles to these predictions to estimate the optimal combination of these feeds for given costs and prices. Feed is the largest variable cost in dairying. The allocation of pasture and supplements that are based on better estimates of milk responses to supplements should lead to increased profit for farmers.

**Abstract:**

Feed is the largest variable cost for dairy farms in Australia, and dairy farmers are faced with the challenge of profitably feeding their cows in situations where there is significant variation in input costs and milk price. In theory, the addition of 5.2 MJ of metabolisable energy to a lactating cow’s diet should be capable of supporting an increase in milk production of one litre of milk of 4.0% fat, 3.2% protein and 4.9% lactose. However, this is almost never seen in practice, due to competition for energy from other processes (e.g., body tissue gain), forage substitution, associative effects and imbalances in rumen fermentation. Pasture species, stage of maturity, pasture mass, allowance and intake, stage of lactation, cow body condition and type of supplement can all affect the milk protein plus fat production response to additional feed consumed by grazing dairy cows. We developed a model to predict marginal milk protein plus fat response/kg DM intake when lactating dairy cows consume concentrates and pasture + forages. Data from peer reviewed published experiments undertaken in Australia were collated into a database. Meta-analysis techniques were applied to the data and a two-variable quadratic polynomial production function was developed. Production economic theory was used to estimate the level of output for given quantities of input, the marginal physical productivity of each input, the isoquants for any specified level of output and the optimal input combination for given costs and prices of inputs and output. The application of the model and economic overlay was demonstrated using four scenarios based on a farm in Gippsland, Victoria. Given that feed accounts for the largest input cost in dairying, allocation of pasture and supplements that are based on better estimates of marginal milk responses to supplements should deliver increased profit from either savings in feed costs, or in some cases, increased output to approach the point where marginal revenue equals marginal costs. Such data are critical if the industry is to take advantage of the opportunities to use supplements to improve both productivity and profitability.

## 1. Introduction

Feed is the largest variable cost for dairy farms in Australia, and dairy farmers are faced with the challenge of profitably feeding their cows in situations where there is significant variation in input costs and milk price [1]. Pasture is generally considered the cheapest source of nutrients [2], and while there is significant variation in the growth rate of pastures throughout the year, farmers can conserve excess pasture as hay and silage to feed back to the herd in times of pasture deficit. Concentrate supplements are also commonly fed to increase stocking rate and overcome deficits in pasture supply [1].

Heard et al. [2] reported new empirical models that predicted the quantitative relationship between milk yield (and milk protein and milk fat yield) and dry matter intake of cereal-based supplements by grazing dairy cows in Australia. Such models are also known as production functions. The models reported by Heard et al. [2] were developed using meta-analysis techniques, and were subsequently employed by Ho et al. [1] to demonstrate the value of applying marginal economic theory to make on-farm, profitable and tactical concentrate feeding decisions. However, as the meta-analysis had only included results from experiments in which grazing cows were fed cereal-based supplements, these models were of limited use because they could not be applied in situations where the cows’ diet also included supplementary hay and silage. While it is difficult to know exactly what proportion of dairy farmers feed their lactating herd both supplementary concentrates and forages as part of the milking ration, on average, hay and silage made up 34% of the total tonnes of DM consumed on the milking area of the farms contributing to the 2019/2020 Dairy Farm Monitor project (C. Waterman pers comm.).

We reasoned that empirical models could be developed that include situations where grazing dairy cows are fed both cereal grain and hay and silage (forage) supplements using meta-analysis techniques. These models would be production functions, which could then be combined with production economics principles to determine the optimal combination of feeds for different on-farm costs and prices. This would support farmers in making more profitable choices between alternative feeds in a tactical setting (weekly, monthly or seasonal timeframe). Here, we report on the meta-analysis and the resulting empirical production function model, and demonstrate the application of production economics to determine the most profitable combination of inputs to feed lactating, grazing dairy cows in southern Australia.

## 2. Materials and Methods

Data from short-term experiments conducted in Victoria that involved dairy cows grazing pasture and fed supplements (both cereal grains and forages) were collated ([3,4,5,6,7,8,9,10,11,12,13,14,15,16,17,18,19,20,21,22,23,24,25,26,27], Table 1). Experiments were included if they met the following criteria: 1. At least two rates of supplement (concentrate and/or forage) were included, 2. For all treatments, the average daily pasture and supplement dry matter intake (DMI) data per cow were available and 3. Daily milk protein and milk fat concentrations and/or yield were measured. In total, this represented 241 lines of data (equivalent to 241 different treatments). A large number of variables and measures from each experiment were included in the database. Their relative contribution to estimates of the marginal milk protein plus milk fat yield response could then be determined via statistical analysis and probabilistic techniques. All variables were included in the database at the outset and disregarded in the statistical analysis as appropriate, rather than collecting an incomplete dataset and overlooking what may be a key variable. The structure of the database allowed for 320 individual parameters to be included, most of which were directly available or calculated from the results of each experiment. This amount of detail allowed a thorough investigation of the factors that most influence marginal milk responses.

The data consisted of treatment means within replicated experiments. Experiments reported in some of the publications had two-factor factorial treatment structures for which only the main effects were reported, since interaction effects were non-significant. In these cases, individual treatment means were estimated, assuming additivity of main effects, in the following way:

(1) $$\mu_{ij}=\mu_{0}+A_{i}+B_{j}$$

where $A_{i}$ is the main effect of level *i* of one factor and $B_{j}$ is the main effect of level *j* of the other factor. This method of predicting treatment means from the reported main effects was employed for each dependent variable, milk yield and milk fat and protein composition and yields.

Individual variation in factors such as seasonal conditions, number of days lactating, body condition score, pasture composition, pasture mass and allowance and amount of supplement consumed by cows meant that no two experiments were the same. The aim of the meta-analysis was to determine the contribution of the input variables to the prediction of milk protein and milk fat yield (kg/cow·day), and to derive predictive equations based on readily available observations of a pasture-based dairy system.

A meta-analysis was performed on the data within the database using a mixed effects, random-coefficients model in which the fixed effects described and tested relationships between covariates and production variables. Relationships between covariates and milk protein and milk fat yield (kg/cow·day) were tested. In this analysis, we aimed to derive a two-variable (bivariate) quadratic relationship between yield (milk protein and milk fat) and concentrate and pasture + forage intake. This was so that the resulting models could be coupled with production economics to help farmers determine the optimal combination of feed inputs for a given situation. A bivariate quadratic equation has the following form:

(2) $$Y=a_{0}+a_{1}X_{1}+a_{2}X_{2}+a_{11}X_{1}^{2}+a_{22}X_{2}^{2}+a_{12}X_{1}X_{2}$$

where

*Y* = yield (milk protein + fat, kg/cow·day)
*X*_1_ = concentrate intake (kg DM/cow·day) and
*X*_2_ = pasture + forage intake (kg DM/cow·day)

The first step in the meta-analysis was data exploration using the Lattice package in R software [28], and subsequently, all models were fitted to data using residual maximum likelihood software in Genstat 18 [29]. Production data (yields of milk protein and fat) were plotted against pasture DMI, forage DMI, concentrate DMI and total DMI, classified by other variables such as experiment, season, liveweight and stage of lactation. Observable trends in the plotted data, along with structural considerations (namely treatment means grouped within experiments), suggested an initial (baseline) mixed model that included linear fixed effects of pasture DMI, forage DMI, concentrate DMI, their interactions with season and random effects consisting of linear, random-coefficients for total DMI within experiments, plus residual variance. The random coefficients allowed for unexplained variation in response to DMI by experiment, as well as encoding the nested (i.e., treatment within experiment) structure of the data. Other fixed-effect terms, such as quadratics separately in pasture DMI, forage DMI and in concentrate DMI, pasture + forage by concentrate DMI cross-product, linear effects of days in lactation, liveweight, pasture nutritive characteristics and groupings of these interacting with DMI variables, were variously added and removed from the baseline model in order to test the significance of associations with the dependent (production) data using analysis of deviance F-tests. The sign and magnitude of estimated coefficients were checked for biological plausibility. Terms with strong associations with the dependent variable were retained in a parsimonious ‘best’ model.

Few experiments in the database had more than two treatment rates of forage DMI, and even fewer with forage DMI in combination with pasture or concentrate rates of DMI. Consequently, the production response to forage DMI, and its interaction with pasture and concentrates (necessary in such models, since the response to one source of DMI depends on the presence and DMI amount from the other sources), was estimated with poor precision. The coefficients of quadratic and cross-product terms in DMI, therefore, were not always estimated to be negative, a requirement for biological plausibility. However, since responses to forage DMI were consistent with those of pasture DMI, a pragmatic solution was to work with the sum, pasture DMI and forage DMI, as a single variable. Henceforth, in this report, ‘pasture + forage’ refers to a variable being the sum of DMIs from these two sources. This constrains the model for pasture and forage responses to follow a common trajectory.

Models for milk protein and fat yields, both individually and as ‘milk solids’ (milk protein plus fat yield), were developed and calibrated separately. This meant that a small degree of inconsistency could be expected. In particular, the sum of yields for milk protein and milk fat individually need not in general equal the same as the results from the direct milk protein plus fat model. This is because a slightly different set of variables can be selected for the different models. There were very small and statistically insignificant differences between the sum of the respective coefficients from the milk protein and milk fat models separately and the milk protein plus fat model. Given this, the simpler milk protein plus fat model was employed. In all cases, predicted values were calculated from the estimated fixed effects only.

The goodness of fit of each model to the data within the database was checked using measures of concordance, Pearson correlation, Lin’s concordance coefficient, Nash–Sutcliffe efficiency coefficient and the root-mean-square error [30,31,32,33].

### Economics

A production function model was developed, relating the DMI of concentrate supplement and pasture + forage with the output of milk protein plus fat. This production function was combined with information on costs (of feeds) and prices received (for milk protein and milk fat), to assess what combination of inputs would be best for the farmer to use to maximise profit [34]. The broader investigation of production functions is called Production Economic Theory, of which Dillon and Hardaker [34] provided a thorough and concise overview.

**Figure 1:**
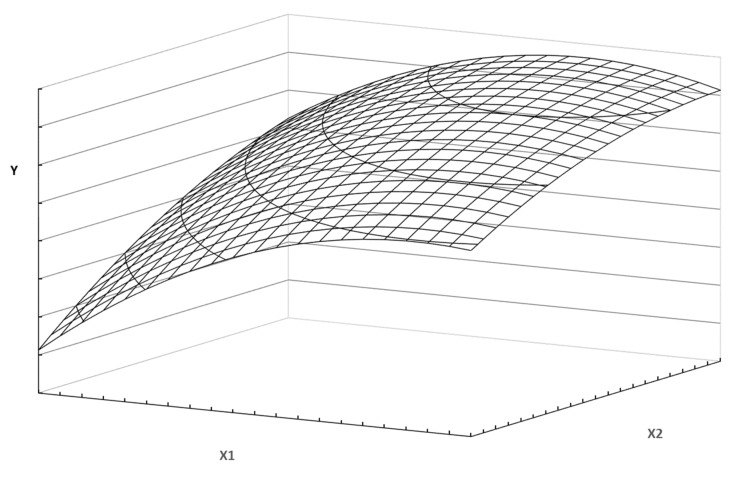
A generic production surface, corresponding to $Y=f\left(X_{1},X_{2}\right)$.

The two variable input production function surface (Figure 1) describes the relationship between the change in quantity of two variable inputs (*X*_1_ and *X*_2_, e.g., concentrates and pasture + forage) and the resulting change in output (y, e.g., milk protein plus fat yield). The height of the surface above any point in the (*X*_1_, *X*_2_) plane shows the amount of output corresponding to that combination of *X*_1_ and *X*_2_ [34].

The primary application of the production function was to estimate the amount of milk protein plus fat output for given quantities of feed input. However, the production function can also be used to determine a number of other key measures. The marginal product, defined as the change in output from an additional unit of feed, was calculated for each input factor [34]. The production function was also used to develop isoquant equations, which describe all the combinations of concentrate and pasture + forage inputs that would yield a specified quantity of milk protein plus fat output. Using the isoquant equations, the rate of technical substitution between inputs could be estimated, identifying the amount by which one variable must be increased if the second variable is decreased by one unit for the level of production to remain the same [34]. The technical substitution rates were, in turn, used to determine the isocline equations, which specify the least cost combination of concentrates and pasture + forage for any feasible amount of output. The final step in our economic analysis was to determine the profit maximising set of inputs, with or without a financial constraint of $3/cow·day. This amount was selected to illustrate application of the marginal economic analysis. The equations for each of these economic analyses are presented (Table 2). It is important to note that the technical substitution between inputs does not factor in biological impacts of changing ratios of feed inputs such as impacts on rumen function. A spreadsheet-based tool was developed to illustrate the production functions and economic concepts. It could also form the foundation for a decision support tool for farmers if the concept was shown to work. Four scenarios were examined to demonstrate these concepts and compare the estimated profit maximising combination of concentrates and pasture + forage in different seasons, with different feed and milk protein and milk fat prices. The input data used for this scenario analysis are given in Table 3. Estimates of pasture intake were necessary and were calculated using the equations published by [35]. Concentrate and forage feed prices represent the 5-year (2014–2018), CPI-adjusted (to 2017/2018 dollars), median values for Gippsland reported by Dairy Australia [36]. Milk protein and milk fat prices represent the CPI-adjusted (to 2019/2020 dollars) seasonal averages across 3 years for a commercial eastern Victorian dairy farm. Levies and charges were not subtracted from these values. An energy-corrected milk composition of 4.0% fat and 3.3% protein was assumed.

## 3. Results

### 3.1. Meta-Analysis

A summary of key animal, pasture and supplementary feed descriptors from experiments included in the database are presented in Table 4.

The best fit, parsimonious model for milk protein + fat was:

(3) $$\begin{array}{cc} Milk\;protein\;+ & fat\;yield\;\left(\mathrm{kg}/\mathrm{cow}.\mathrm{day}\right)\\ & =\mu +\delta x_{SY}+\theta x_{Week}+\alpha_{p+f}x_{p+f}+\beta_{p+f}x_{p+f}^{2}+\alpha_{c}x_{c}+\beta_{c}x_{c}^{2}\\ & +\gamma x_{P+fc}+\tau_{Season}+\alpha_{c.Season}x_{c}+\lambda_{LWT}+\vartheta x_{DMD\%}+E_{i}+B_{i}x_{DMI}\\ & +\varepsilon_{ij} \end{array}$$

Each covariate was centred, that is, *x* = covariate-mean (covariate). The $E_{i}$ and $B_{i}$ represent bivariate normal random mean and slope coefficients in total DMI, for experiment *i*. The residual error for the datum *j* of experiment *i* is denoted $\varepsilon_{ij}$. These models can be manipulated so that the direct coefficients for the bivariate quadratic equation form can be determined.

Definitions of covariates, x, coefficients and standard errors (Table 5) and means of covariates are provided below (Table 6).

Using data from Table 5 and Table 6, the significant equation for milk protein + fat yield (kg/cow·day) can, therefore, be written as:

(4) Milk protein + fat (kg/cow·day) = 1.465 + 0.178 × (pre-experimental milk protein + fat yield − 1.63) − 0.006 × (weeks lactating − 18.18) + season (Spring = 0, Summer = −0.174, Autumn = −0.312) + 0.100 × (DMI pasture + forage − 12.82) + 0.107 × (DMI conc − 2.26) − 0.002 × (DMI pasture + forage^2^ − 172.90) − 0.005 × (DMI concentrates^2^ − 11.49) − 0.002 × (DMI pasture + forage x concentrates − 26.13) + Season × DMI concentrates (Spring = 0, Summer = 0.020, Autumn = 0.030) + Liveweight group (<500 kg = 0, >500 kg = 0.030) + 0.014 × (Past DMD% consumed − 72.63)

**Figure 2:**
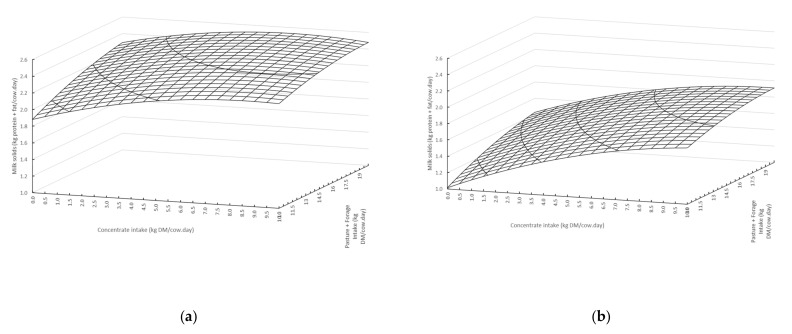
Production surface curves for the two-variable quadratic relationship between concentrate and pasture + forage intake and predicted milk protein plus fat yield (kg/cow·day) for the (**a**) spring and (**b**) autumn scenarios.

Milk protein plus fat yield was lower in summer (−0.17 kg/cow·day) and autumn (−0.31 kg/cow·day) than in spring. However, there was a positive interaction with concentrate intake and season, with greater milk protein plus fat yield response to concentrates in summer (0.02 kg/cow·day) and autumn (0.03 kg/cow·day) than spring. Milk protein plus fat yield was also strongly related to DMI of concentrate and pasture + forage supplement in that each had significant linear and quadratic terms. The quadratic coefficient estimates for pasture + forage and concentrate DMI were negative, consistent with a diminishing milk protein plus fat yield response as DMI increases. Cows heavier than 500 kg liveweight were determined to produce more milk protein plus fat than cows less than 500 kg liveweight (a difference of 0.01 kg/cow·day). Finally, the digestibility of pasture consumed was significant, with higher digestibility pasture leading to increased yields of milk protein plus fat. Production surfaces for the two-variable quadratic relationship between concentrate and pasture + forage DMI and milk protein plus fat yield for spring and autumn are presented (Figure 2).

How well the model fit the data within the database—the ‘goodness of fit’—was tested using measures of concordance (Table 7).

The fitted model for milk protein plus fat yield was shown to closely reflect milk protein plus fat yield measured under experimental conditions (r = 0.93). However, this is to be expected, as the same data used to build the models were also used in this instance to test the ‘goodness of fit’. Ideally, the models need to be tested against ‘novel’ data—data that has not contributed to the meta-analysis. Such data are currently lacking, and we did not have enough data in our dataset to hold some back for this purpose.

### 3.2. Economic Analysis

The predicted profit maximising combination of concentrates and pasture + forage with respect to milk protein plus fat yield (kg/cow·day) was calculated for the scenarios presented in Table 3 (Table 8).

**Figure 3:**
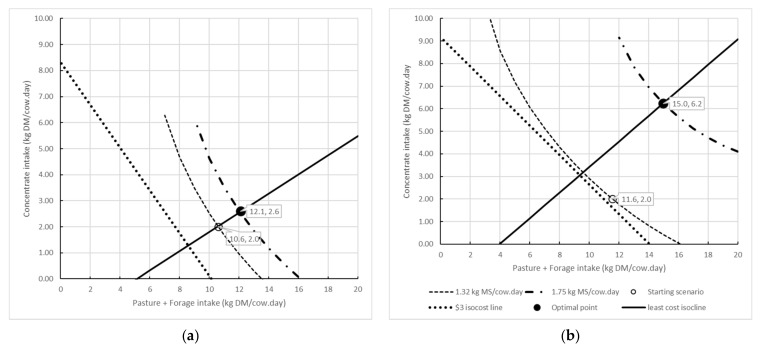
Isoquants representing the starting scenario (---) and optimal input combination (**-·-·-·**), least cost isocline (―), isocost line with $3/cow·day financial constraint (····), starting scenario (o) and optimal input combination (•) for the (**a**) spring and (**b**) autumn scenarios.

Seasonal fluctuations in milk protein plus fat and feed prices, together with changes in animal physiology with the progression of lactation, led to variable responses, and therefore, variable profit maximising combinations of inputs. For the spring scenario, for the given combination of costs and prices, it was predicted that DMI of concentrates be increased to 2.6 kg DM/cow·day from 2 kg/cow·day, and pasture + forage DMI increased by 1.5 kg DM to 12.1 kg DM/cow·day (Table 8, Figure 3a), which would lead to an increase in milk protein plus fat yield from 2.1 to 2.2 kg/cow·day. This would cost $4.49/cow·day, and return $13.42/cow·day, generating a profit of $8.93/cow·day. By contrast, under the autumn scenario, the profit maximising amount of concentrate (6.2 kg DM/cow·day) and pasture + forage (15.0 kg DM/cow·day; Table 8, Figure 3b) was predicted to generate 1.8 kg of milk protein plus fat/cow·day. This would cost $5.23 and return $12.60/cow·day; a profit of $7.37/cow·day.

For each of the scenarios, isoquants, least cost isoclines, optimal input combinations and isocost combinations in the face of financial constraint were calculated. For brevity, the spring and autumn scenarios are presented (Figure 3).

**Figure 4:**
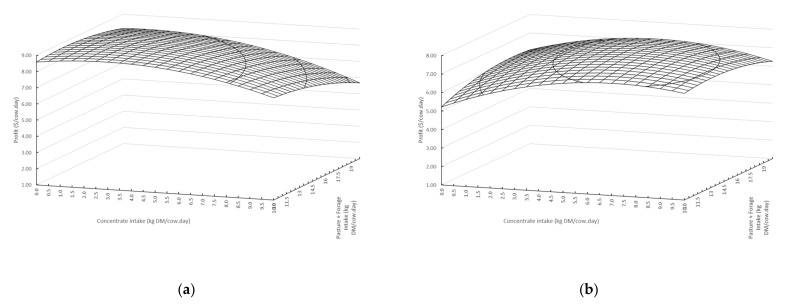
Profit surface curves for the two-variable quadratic relationship between concentrate and pasture + forage intake and predicted profit ($/cow·day) for the (**a**) spring and (**b**) autumn scenarios.

These isoquants represent all the combinations of concentrate and pasture + forage for a given level of milk protein plus fat output for both the starting scenario and the modelled optimal input combination. Isoclines denote the least cost combination of inputs for a specified quantity of output, based on the unit price of concentrates and pasture + forages. Logically, the point at which the isocline transects any isoquant represents the least cost combination of feeds for the given output. In the situation where there is no constraint on the quantity of outputs to be produced, or on the quantity of inputs available [33], the profit maximising combination of inputs can be calculated, taking into account both the price paid for inputs and the price received for product. The isocost line describes the various combinations of concentrate and pasture + forage in the situation of a financial constraint. The point at which the isocost and isocline lines intercept represents the least cost combination of inputs for the given financial constraint, which was set at $3/cow·day. For the spring scenario, this is predicted to be 8.6 kg DMI of pasture + forage and 1.3 kg of concentrate supplement, and for the autumn scenario, 9.4 kg DMI of pasture + forage and 3.1 kg DMI of concentrate supplement. Milk protein plus fat output for this scenario could then be calculated. The shape of the production surfaces for the two-variable quadratic relationship between concentrate and pasture + forage DMI and profit (total milk income minus the total feed cost) for the spring and autumn scenarios are presented in Figure 4. The production surfaces demonstrate the different combinations of feed inputs to give the same profit.

## 4. Discussion

Currently, farmers make tactical decisions about how much supplement to feed implicitly. Many strategies are employed; some farmers feed supplements according to current milk production and changes they expect, some according to stage of lactation, some use a strategy of flat-rate feeding and some aim to manage their pastures to a consistent grazing height and use indicators such as overgrazing or wastage to judge the appropriate rate of supplement to feed [1]. However, to profitably feed supplements, it is important that farmers know how much extra milk of a particular composition will be produced for each kilogram of supplement consumed [2]—that is, the immediate marginal milk response. This knowledge can be coupled with the milk price received to determine the most profitable level of supplements to feed, i.e., the point at which marginal revenue from the extra milk just exceeds the marginal cost of the extra feed. Allocating supplements based on well-informed and more accurate estimates of marginal milk responses to supplements has the potential to improve farm profit by reducing feed costs or increasing the amount of profitable output [1].

There are numerous and complex interactions that influence the immediate marginal milk response to supplements. Factors such as season, the nutritive characteristics of pastures, pregrazing pasture mass and allowance of pasture on offer [4,15,18,37], amount of pasture consumed [4,18], amount and type of supplement consumed, nutritive characteristics of the supplement [4,5,18,23], amount of substitution [15,18] and animal and management factors, such as stage of lactation [5], body condition score [19], frequency of feeding [38] and genetic merit of cows [39], have all been shown to influence milk production when supplements are fed.

In the present study, a newly generated response function of milk protein plus fat yield was developed and used to analyse the economics of tactical feeding decisions where both grain and forage supplements are fed. The question examined was how much supplement plus pasture should be fed over a short time period, such as the next fortnight, to maximise profit given a particular farm situation, incorporating information such as starting milk yield and stage of lactation and what is known about milk and supplementary feed prices. It was also demonstrated how the response function could be used to determine the marginal product, the rate of technical substitution between inputs and to generate isoquants. The response function described here builds on the work reported by [1,2], which described a response function for grazing cows fed concentrate supplements only.

The idea of developing a method of predicting the immediate marginal milk response is not new. Ideally, a mechanistic model that incorporates concepts about underlying biology [40] would be available to predict milk solids production when concentrates and pastures + forages are fed. However, it has been shown that for grazing cows, predicted immediate milk responses using commercially available, mostly mechanistic models are often in disagreement. Testing 11 of the most commonly used programs, Little et al. [41] reported that the predicted immediate milk response from an additional 5 kg DM of cereal grain ranged from 1.0 to 14.0 L of milk, corresponding to immediate marginal milk responses of between 0.2 and 2.8 kg milk/kg DM cereal grain. This wide range in predicted responses illustrates the difficulty in modelling complex biological systems.

The new empirical model described here was developed from a dataset of experiments offering concentrates and pasture + forage to lactating dairy cows over several decades. The objective of the work was to develop a model that would have easy, on-farm application, drawing on experimental data, without detailed metabolic measurements. Thus, an empirical, predictive approach, requiring uncomplicated inputs was essential. This model has application within boundaries largely defined by the nature of the research used to build it. The model was built using data from short-term experiments, where cows grazing temperate pastures were supplemented with cereal-based or forage supplements. It is, therefore, recommended that this model is only applied in situations such as the one described. The milk response to supplements in primiparous cows could be expected to be less than for multiparous cows; however, from the current dataset, there were insufficient data to distinguish the differences between the two. In the present analysis, milk yields from animals lighter than 400 and heavier than 600 kg are largely untested, as are milk yields from cows beyond 270 days in milk. The influence of the genetic merit of cows [38] is also not captured in this model. Empirical models of this kind, developed on, and calibrated to, a wide diversity of experimental data collated from different locations in different decades, cannot detect and reliably represent the minutiae of biological processes. However, they may detect and summarise the major processes and represent an average response that may be expected to apply, at least approximately, over a wide set of conditions.

Yields of milk protein and milk fat from dairy cows are strongly positively correlated with milk yield [42]. However, while total yields of protein and fat in general increase with increasing milk yield, the concentration of these components often decreases. Milk protein concentration and yield can be altered via dietary manipulation, by increasing the overall energy intake with a concurrent reduction in the pasture to concentrate ratio [43]. Decreasing the pasture-to-concentrate ratio has also been shown to reduce milk fat concentration, as has increasing overall DMI [44]. The amount of physically effective fibre, the composition of the carbohydrate within the concentrate being fed, lipid intake and frequency of feeding have also been shown to affect milk fat concentration [44]. In our model, DMI of pasture + forage, or of concentrate, significantly increased milk protein + fat yield, and would be expected to increase milk yield. These both had negative quadratic coefficient estimates, consistent with a diminishing milk protein + fat yield response as DMI increases. However, in the model presented here, it is difficult to uncouple the impacts of changes in milk protein and milk fat concentration and changes to milk protein and fat yield. It is also difficult to categorically uncouple the impacts of season main effects and stage of lactation, as the majority of cows contributing to the database of experimental results calved in spring. The season factor in the model serves to take out some of the between-experiment variation, as well as being necessary before considering DMI interactions with season. Importantly, the information on responses to DMI, which comes mostly from within rather than between experiments, are more reliable, and was the primary focus of this work.

A necessary simplifying assumption was to use the sum of pasture and forage DMI as a single variable in the response function. This was because there were few experiments available that had more than two treatment rates of forage DMI or forage DMI in combination with different rates of pasture or concentrate DMI. If additional data became available, the meta-analysis could be revised to develop a three-variable response function where concentrate, forage and pasture DMI were separate inputs. This would also enable pasture and forage supplements to be valued separately in the economic analysis rather than treated as substitutes as done in this study [45].

Although the response function used and the analysis performed accounted for the contribution of supplement + pasture to milk protein plus fat production only, there are benefits of feeding concentrates to replenish body tissue, increasing body condition and improving reproduction and animal health. The potential of these longer-term benefits means that farmers will continue to feed supplements when it may appear to be uneconomic on a ‘milk only’ basis.

## 5. Conclusions

We developed a model that could be used to predict marginal milk protein plus fat response/kg DM intake when lactating dairy cows consume concentrates and pasture + forages. Data from peer reviewed published experiments undertaken in Australia were collated into a database. Meta-analysis techniques were applied to the data and a two-variable quadratic polynomial production function was developed, based on relatively simple inputs. Production economic theory was used to estimate the level of output for given quantities of input, the marginal physical productivity of each input, the isoquants for any specified level of output and the optimal input combination for given costs and prices of inputs and output.

Limitations to the application of this on-farm work predominantly lie with the scarcity of appropriate data with which to develop the model. However, given feed accounts for the largest input cost in dairying, allocation of pasture and supplements that are based on better estimates of marginal milk responses to feed inputs should deliver increased profit from either savings in feed costs, or, in some cases, increased output to approach the point where marginal revenue equals marginal costs. Such data are critical if the industry is to take better advantage of the opportunities to use supplements to improve both productivity and profitability. If the model is to be further developed and used in farmer decision tools, it will be important to test whether it can be used to predict novel production data, how it compares with commercially available computer models and also the sensitivity of predictions to changes in key inputs.

## Figures and Tables

**Table 1:** Summary of experiments contributing to the meta-analysis.

Ref. No.	Description of Experiment	Treatments	Breed	LW	DIM	Season	Milk Yield (kg/cow·day)	Milk Fat (%)	Milk Protein (%)
[3]	Hay supplementation on a restricted intake of paspalum pasture. Two experiments with target pasture:hay intake ratios	PO, 75P:25H, 50P:50H, 75P:25H, 50P:25H, 50P	J × F	405	240	Autumn	11.2		
		PO, 75P:25H, 50P:50H, 75P:25H, 50P:25H, 50P	J × F	396	270	Autumn	7.6		
[4]	Level of concentrate feeding and pasture allowance on productivity of cows in late lactation	2 × pasture allowance, 4 × concentrate supplement intake	J + F	427	240	Autumn	10.3	5	3.5
[5]	Stall-fed cows fed a basal ration of pasture supplemented with varying amounts of pelleted concentrate supplement	PO, P + 1.8C, P + 2.7C, P + 5.4C, P + 9.6C	n/d	459	29	Spring	25.3	4.1	3.3
		PO, P + 1.8C, P + 3.6C, P + 6.1C	n/d	475	205	Spring	13.8	4.9	3.6
		PO, P + 3.6C, P + 8.7C	n/d	450	81	Spring	19.1	3.8	3.3
		PO, P + 2.2C, P + 4.4C	n/d	431	224	Spring	11.1	5.2	4
		PO, P + 2.2C, P + 4.5C	n/d	422	58	Spring	19.2	4.7	3.5
[6]	Pasture substitution rates with variable pasture allowances	LA PO, LA + C, MA PO, MA + C, HA PO, HA + C	F, F × J	454	21	Spring	19	4.3	3.2
[7]	Level of pasture feeding on milk responses to high energy supplements	LA + 0, LA + 2.2C, LA + 4.5C, MA + 0, MA + 2.2C, MA + 4.5C, HA + 0, HA + 2.2C, HA + 4.5C	F × J	434	210	Spring	11.6		
		LA + 0, LA + 2.2C, LA + 4.5C, MA + 0, MA + 2.2C, MA + 4.5C, HA + 0, HA + 2.2C, HA + 4.5C	F × J	426	60	Spring	19.7		
[8]	Influence of high energy supplements on the productivity of pasture-fed cows	PO, P + 3.3C, P + 3.8C + FA	J × F	505	35	Winter	22.8	4.4	2.9
[9]	Grazing cows supplemented with 3 or 8 kg DM/cow·day maize silage	HA P, HA P + 3 kg MS, HA P + 3 kg MS + prot, LA P + 8 kg MS, LA P + 8 kg MS + prot	F	424	38	Spring	20.8		
[10]	Milk responses of cows to Persian clover and maize silage	Clover, Clover + 4 kg MS, Clover + 8 kg MS	n/d	500		Spring	19		
[11]	Productivity of cows grazing white clover supplemented with maize silage	PO, P + 4–5 kg MS	F	~500	variable	variable	14.8–29.1	3.9–5.0	2.8–3.5
[12]	Productivity of cows grazing white clover supplemented with maize silage	LA, LA + 4.4 MS, HA, HA + 4.4 MS	F	498	213	Autumn	14.9	4.3	3.1
[13]	Milk production responses when cows grazing either paspalum or white clover pastures are fed supplements	Pas, Pas + MS, Pas + MS + B, Pas + MS + CSM; Clo, Clo + MS, Clo + MS + B, Clo + MS + CSM	F	507	234	Autumn	15.4	4.2	3.2
[14]	Influence of energy and protein supplements on the productivity of cows grazing white clover swards	PO, P + 5 MS, P + 3 MS + 2 B, P + 3 MS + 2 CSM, P + 3 MS + 1 B + 1 CSM	F	500	110	Spring	28.2	4.5	3.1
		PO, P + 5 MS, P + 3 MS + 2 B, P + 3 MS + 2 CSM, P + 3 MS + 1 B + 1 CSM	F	519	154	Summer	24.2	4.2	3.1
[15]	Responses to grain feeding by grazing dairy cows	LA, LA + 5C, HA, HA + 5C	F, F × J	510	180	Autumn	17.6	4.2	3.2
		PO, P + 2C, P + 4C, P + 8C	F, F × J	534	180	Autumn	21.4	4.3	3.1
[16]	Length of the period of supplementation with concentrates on pasture intake and milk production of grazing cows	PO, P + 5C	n/d	552	142	Spring	26.4	4.1	3
		PO, P + 5C	n/d	560	194	Summer			
		PO, P + 5C	n/d	581	243	Autumn			
[17]	Effect of cereal grain, lupins-cereal grain or hay supplements on the intake and performance of grazing cows	PO, P + 5C, P + 5 C/L, P + 5 H	F	548	105	Spring	30	4.1	3.2
		PO, P + 5C, P + 5 C/L, P + 5 H	F	550	114	Summer	25.6	3.8	2.8
		PO, P + 5C, P + 5 C/L, P + 5 H	F	545	222	Autumn	16.9	4.6	3.5
[18]	Effects of variation in herbage mass, allowance and level of supplement on milk production	LM LA, LM LA + C, LM HA, LM HA + C, HM LA, HM LA + C, HM HA, HM HA + C	HF	582	126	Summer	25.2	3.5	2.9
[19]	Low and high body condition score or larger and smaller body size and low and high pasture allowance with or without supplements	LBC LA PO, LBC LA + C, LBC HA PO, LBC HA + C	F	476	22	Spring	25.8	3.8	3.0
		HBC LA PO, HBC LA + C, HBC HA PO, HBC HA + C	F	551	19	Spring	24.9	3.9	3.0
		LBS LA PO, LBS LA + C, LBS HA PO, LBS HA + C	F	486	40	Summer	28.4	4.0	3.0
		HBS LA PO, HBS LA + C, HBS HA PO, HBS HA + C	F	618	51	Summer	31.8	3.8	2.9
[20]	Effects of feeding additional pasture hay in autumn to grazing cows supplemented with barley grain	PO, P + 6C, P + 6C + 0.5H, P + 6C + 1.2H, P + 6C + 2H, P + 6C + 3H	F	578	172	Autumn	19.7	4.1	3.1
[21]	Effect of grain supplementation on milk production	PO, P + 6C	HF	521	29	Spring	27	3.8	3.5
[22]	Effect of grain supplementation and chemical or physical fibre on marginal milk responses of grazing cows	LA, HA, LA + 2.5 FP, LA + 2.5 FC, LA + G, LA + 7.5 G/HP, LA + 7.5 G/HC	F	520	49	Spring	25.2	4.1	3
[23]	Effect of grain supplementation on milk response in mid-lactation grazing dairy cows	PO, P + 3C, P + 5C, P + 7C, P + 9C, P + 11C	HF	549	167	Autumn	22.3	3.9	3.3
		PO, P + 3C, P + 5C, P + 7C	HF	544	166	Autumn	194	4.1	3
[24]	Effect of grain and straw supplementation on marginal milk production responses	PO, P + 0.5S, P + 1.0S, P + 2.0S, P + 0S + 5, P + 0.5S + 5, P + 1.0S + 5, P + 2.0S + 5	HF	588	43	Spring	29.9	4	3.1
[25]	Effect of grain and fibre supplements on milk production responses	PO, P + 5C, P + 1.8S, P + 5C + 1.8S	F	535	31	Spring			
[26]	Effect of feeding an energy supplement with white clover silage	WCS, WCS + 4.5C	HF	550	90	Summer	19.6	4	3
[27]	Effect of increasing amounts of crushed wheat fed to cows consuming Persian clover	PO, P + 1.2C, P + 2.6C, P + 3.5C, P + 5.3C	HF	550	32	Spring	33.3	4.1	3.2

PO = pasture only, P = pasture, H = hay, S = straw, MS = maize silage, B = barley, CSM = cotton seed meal, C/L = cereal grain/lupins, C = concentrate supplement, WCS = white clover silage, LA = low pasture allowance, MA = medium pasture allowance, HA = high pasture allowance, FA = fatty acid, LM = low pasture mass, HM = high pasture mass, LBC = low body condition score, HBC = high body condition score, LBS = low body size, HBS = high body size, FP = fibre pellet, FC = fibre cube, G/HP = grain/hay pellet, G/HC = grain/hay cube, J = Jersey, F = Friesian, n/d = not defined. Milk yield, milk fat (%) and milk protein (%) reflect the pre-experimental average of all cows in the experiment.

**Table 2:** Equations for calculating economic measures based on a two-variable quadratic production function with the form: $Y=a_{0}+a_{1}X_{1}+a_{2}X_{2}+a_{11}X_{1}^{2}+a_{22}X_{2}^{2}+a_{12}X_{1}X_{2}$. Taken from [34].

Economic Measure	*X*_1_	*X*_2_
Marginal Product: The change in output from using an additional unit of one input, holding the second input constant.	Marginal product of *X*_1_ a_1_ + 2a_11_*X*_1_ + a_12_*X*_2_	Marginal Product of *X*_2_ a_2_ + 2a_22_*X*_2_ + a_12_*X*_1_
Isoquant: describes all combinations of inputs which yield a specified quantity of output	*X*_1_ = {−(a_1_ + a_12_*X*_2_) + [(a_1_ + a_12_*X*_2_)^2^ − 4a_11_(a_2_*X*_2_ + a_22_*X*_2_^2^ + a_0_ − Y)]^1/2^}/2a_11_	*X*_2_ = {−(a_2_ + a_12_*X*_1_) + [(a_2_ + a_12_*X*_1_)^2^ − 4a_22_(a_1_*X*_1_ + a_11_*X*_1_^2^ + a_0_ − Y)]^1/2^}/2a_22_
Rate of technical substitution: the amount by which one input must be increased if the second input is decreased by one unit and the level of production is to be maintained.	−(a_2_ + 2a_22_*X*_2_ + a_12_*X*_1_)/(a_1_ + 2a_11_*X*_1_ + a_t2_*X*_2_)	−(a_1_ + 2a_11_*X*_1_ + a_t2_*X*_2_)/(a_2_ + 2a_22_*X*_2_ + a_12_*X*_1_)
Least cost isocline: the least cost combination of the two inputs for the production of any specified quantity of output.	At every point along the least cost isocline, the rate of technical substitution (RTS) of *X*_1_ for *X*_2_ is inversely equal to the negative ration of their prices (P_1_ = price of *X*_1_, P_2_ = price of *X*_2_): −RTS_12_ = P_2/_P_1_ = k The solution of this equation gives the least cost isocline for a two-variable quadratic as: *X*_1_ = [ka_1_ − a_2_ + (ka_12_ − 2a_22_)*X*_2_]/(a_12_ − 2 ka_11_)	At every point along the least cost isocline, the rate of technical substitution (RTS) of *X*_2_ for *X*_1_ is inversely equal to the negative ration of their prices (P_2_ = price of *X*_2_, P_1_ = price of *X*_1_): −RTS_21_ = P_1/_P_2_ = k The solution of this equation gives the least cost isocline for a two-variable quadratic as: *X*_2_ = [ka_2_ − a_1_ + (ka_12_ − 2a_11_)*X*_1_]/(a_12_ − 2 ka_22_)
Optimal input combination: The profit maximising combination of inputs, assuming there are no constraints on the quantity of output produced.	*X*_1_ = (p_1_/p_y_ − a_1_ − a_12_*X*_2_)/2a_11_	*X*_2_ = (p_2_/p_y_ − a_2_ − a_12_*X*_1_)/2a_22_

**Table 3:** Input data used to demonstrate milk response functions to concentrate and pasture + forage intake.

Season	Spring	Summer	Autumn	Winter
Dominant pasture species	Perennial Ryegrass	Perennial Ryegrass	Perennial Ryegrass	Perennial Ryegrass
Pasture mass (kg DM/ha)	3183	3891	3905	2668
Area (ha)	1.5	1.5	1.5	1.5
Number of cows	200	200	200	200
Pasture height (cm)	7	7	8	4
Current milk production (kg/cow·day)	28	23	16	10
Current milk fat (%)	3.92	4.22	4.80	4.95
Current milk protein (%)	3.47	3.13	3.42	3.72
Current milk protein plus fat production (kg/cow·day)	2.07	1.69	1.32	0.87
Number of weeks lactating	8	19	28	42
Average liveweight (kg)	500	500	500	500
Current dry matter intake concentrate (kg DM/cow·day)	2	2	2	2
Current dry matter intake forage supplement (kg DM/cow·day)	0	0	0	0
Estimated pasture dry matter intake (kg DM/cow·day) *	10.6	11.4	11.6	8.9
DM content concentrate supplement (%)	90	90	90	90
DM content forage supplement (%)	85	85	85	85
Metabolisable energy of pasture consumed (MJ/kg DM)	11.94	9.93	10.07	11.12
Concentrate feed price ($/tonne delivered)	324	300	294	314
Forage feed price ($/tonne delivered)	251	204	182	217
Milk protein price ($/kg milk protein)	7.99	9.14	9.93	9.67
Milk fat price ($/kg milk fat)	4.65	5.02	4.95	4.74

* Pasture DMI is estimated based on equations published by [34].

**Table 4:** Average, minimum, maximum and standard deviation of key animal, pasture and supplementary feed descriptors included in the database.

	Average	Minimum	Maximum	Standard Deviation
Liveweight (kg)	508	396	618	56.5
Standard Reference Weight (kg)	520	403	635	62.0
Days in Milk (d)	134	18	270	79.1
Pre-experimental milk production (kg)	21.0	7.6	34.8	6.38
Pre-experimental energy-corrected milk (kg/cow·day)	21.5	11.7	35.7	5.37
Milk fat (g/100 g)	4.19	3.51	5.20	0.360
Milk protein (g/100 g)	3.17	2.76	4.00	0.219
Body condition score at start of experiment (scale 1–8)	4.17	3.25	5.00	0.384
Pasture allowance (kg DM/cow·day)	28.1	0	52.6	11.14
Dry matter intake—pasture (kg/cow·day)	11.4	0	20.9	3.33
In vitro dry matter digestibility pasture consumed (% DM)	72.7	58.7	85.4	7.51
Crude protein pasture consumed (% DM)	19.2	7.9	34.2	5.07
Neutral detergent fibre pasture consumed (% DM)	45.9	29.5	64.8	11.58
Dry matter intake—concentrate supplement (kg/cow·day)	2.2	0	10.4	2.47
Metabolisable energy concentrate consumed (MJ/kg DM)	12.4	9.9	14.0	0.78
Crude protein concentrate consumed (% DM)	15.6	9.4	63.5	7.80
Neutral detergent fibre concentrate consumed (% DM)	18.5	11.3	34.0	4.46
Dry matter intake—forage supplement (kg/cow·day)	2.6	0	17.7	3.12
Metabolisable energy forage consumed (MJ/kg DM)	9.4	4.6	11.0	1.22
Crude protein forage consumed (% DM)	9.3	5.6	19.3	3.86
Neutral detergent fibre forage consumed (% DM)	52.3	33.6	70.4	9.53

**Table 5:** Coefficients and standard errors (s.e.) for covariates included in the model for milk protein plus fat yield (kg/cow·day).

Term/Covariate		Milk Protein Plus Fat Yield	
		Coefficient	s.e.
Constant	μ	1.465	0.0352
Pre-experimental covariate ^†^	δ	0.178	0.0790
Weeks lactating	θ	−0.006	0.0024
DMI * pasture + forage	$\alpha_{P+f}$	0.100	0.0189
DMI * pasture + forage squared	$\beta_{P+f}$	−0.002	0.0007
DMI * concentrate	$\alpha_{c}$	0.107	0.0168
DMI * concentrates squared	$\beta_{c}$	−0.005	0.0011
DMI * pasture + forage×DMI * concentrate	γ	−0.002	0.0011
Season—Winter/Spring	$\tau_{Spr}$	0.000	
Season—Summer	$\tau_{Sum}$	−0.174	
Season—Autumn	$\tau_{Aut}$	−0.312	
DMI * concentrate × Season—Spring	$\alpha_{c.Spr}$	0.000	
DMI * concentrate × Season—Summer	$\alpha_{c.Sum}$	0.020	
DMI * concentrate × Season—Autumn	$\alpha_{c.Aut}$	0.030	
Liveweight group (<500 kg)	ʎ $LWT_{i}$	0.000	
Liveweight group (>500 kg)	ʎ $LWT_{ii}$	0.116	
Pasture dry matter digestibility (%, consumed)	ϑ	0.014	0.0022

^†^ Pre-experimental covariate = the dependent variable (average milk or milk protein plus fat yield) measured during a pre-experimental covariate period. * DMI = dry matter intake. LWT = liveweight.

**Table 6:** Means of covariates from the final model for milk protein plus fat yield (kg/cow·day).

Covariate		Milk Protein + Fat
Pre-experimental covariate ^†^	$\;x_{SY}$	1.63
Weeks lactating	$x_{Week}$	18.18
DMI * pasture + forage	$x_{p+f}$	12.82
DMI * pasture + forage squared	$x_{p+f}^{2}$	172.90
DMI * concentrates	$x_{c}$	2.26
DMI * concentrates squared	$x_{c}^{2}$	11.49
DMI * pasture + forage × DMI concentrates	$x_{cp+f}$	26.13
Pasture dry matter digestibility (%, consumed)	$x_{\vartheta }$	72.63

^†^ Pre-experimental covariate = the dependent variable (average milk protein plus fat yield) or measured during a pre-experimental covariate period. * DMI = dry matter intake.

**Table 7:** Measures of fit for the model determined for milk protein plus fat yield (kg/cow·day). The comparison is made between yields measured experimentally and yields predicted employing the fitted model fixed effects. n = number of treatment means contributing to model, r = correlation coefficient, Lin’s = Lin’s concordance coefficient, RMSE = Root Mean Square Error of difference and NSE = Nash–Sutcliffe coefficient of efficiency.

Model	n	r	Lin’s	RMSE	NSE
Milk protein plus fat yield (kg/cow·day)	241	0.928	0.927	0.161	0.852

**Table 8:** Predicted profit maximising combination of inputs (concentrates and pasture + forage ± standard error) for milk protein plus fat yield, for each season, based on modelled scenarios.

Season	Concentrates (kg DM/cow·day)	Pasture + Forage (kg DM/cow·day)	Predicted Milk Protein Plus Fat Yield (kg/cow·day)	Feed Costs ($/cow·day)	Milk Income ($/cow·day)	Predicted Profit ($/cow·day)
Spring	2.6 (±3.11)	12.1 (±4.63)	2.2	4.49	13.42	8.93
Summer	5.2 (±2.84)	14.2 (±4.37)	2.0	5.12	13.91	8.79
Autumn	6.2 (±2.75)	15.0 (±4.34)	1.8	5.23	12.60	7.37
Winter	6.1 (±2.88)	13.3 (±4.50)	1.4	5.51	9.69	4.18

## Data Availability

Publicly available data sets were analysed in this study, and these have been referenced in the manuscript.
